# The association between admission serum albumin and preoperative deep venous thrombosis in geriatrics hip fracture: a retrospective study of 1819 patients with age ≥ 65 years

**DOI:** 10.1186/s12891-023-06776-1

**Published:** 2023-08-24

**Authors:** Yi-Lun Wu, Dan Zhang, Kai-Yuan Zhang, Ting Yan, Wen-Si Qiang, Ting Zhang, Bin-Fei Zhang

**Affiliations:** 1 Department of Ultrasound Medicine, Honghui Hospital, Xi’an Jiaotong University, Beilin District, Xi’an, Shaanxi Province China; 2 Department of Joint Surgery, Honghui Hospital, Xi’an Jiaotong University, No. 555 Youyi East Road, Xi’an, 710054 Shaanxi Province China

**Keywords:** Hip fracture, Albumin, DVT, Risk, Complication

## Abstract

**Objective:**

This study evaluated the association between serum albumin levels and preoperative deep vein thrombosis (DVT) in geriatric hip fractures.

**Methods:**

Older adult patients with hip fractures were screened between January 2015 and September 2019. The demographic and clinical characteristics of the patients were collected. Multivariate binary logistic regression and generalized additive model were used to identify the linear and nonlinear association between albumin levels and preoperative DVT. Analyses were performed using EmpowerStats and the R software.

**Results:**

A total of 1819 patients were included in this study. The average age was 79.37 ± 6.88 years. There were 550 males and 1269 females. The preoperative albumin was 38.19 ± 4.07 g/L. There were 580 (31.89%) preoperative DVTs. Multivariate binary logistic regression showed that albumin level was associated with preoperative DVT (odds ratio [OR] = 0.94, 95% confidence interval [CI]: 0.91–0.97, *P* = 0.0002) after adjusting for confounding factors. The fully adjusted model showed a DVT risk decrease of 6% when albumin concentration increased by one g/L after controlling for confounding factors. In addition, the trend test and propensity score matching also showed a stable linear correlation between albumin level and preoperative DVT.

**Conclusion:**

Serum albumin is associated with preoperative DVT in geriatric patients with hip fractures, and it could be considered a predictor for the risk of DVT.

**Registration ID:**

ChiCTR2200057323.

**Supplementary Information:**

The online version contains supplementary material available at 10.1186/s12891-023-06776-1.

## Introduction

Hip fracture in the elderly has attracted extensive attention due to its epidemiological characteristics. Its incidence is high. Even studies in China and South Korea showed an upward trend [1, 2]. A study from China reported that the incidence of hip fractures over 60 years of age increased from 2007 (63.57%) to 2018 (74.05%) [1]. The high mortality rate associated with hip fractures has been well documented in studies and surveys in the United States and Europe [3–5]. It was reported that the one-year mortality was 30% rate after hip surgery [4]. Finally, it impairs patients’ long-term quality of life and increases the economic burden on families and even the whole society [6, 7].

Deep vein thrombosis (DVT) is a common complication of fracture. It can have many adverse effects, such as pulmonary embolism, edema of the injured limb, superficial varicose veins, and secondary ulceration or necrosis. Among patients with hip fractures, elderly patients were more likely to develop DVT, with incidence rates ranging from 16.3–66.7%[8–11]. Recently, many risk factors have been confirmed to be closely related to DVT after hip fracture. For example, Song et al. showed that long immobilization duration and increased D-dimer levels were associated with preoperative DVT independently [11]. Another study identified risk factors including age, female sex, time waiting for surgery, and time from injury to admission, in addition to confirming the previous study [10]. Other factors included kidney failure, recent surgery, smoking, clinical signs, and coronary heart disease (CHD) [8, 9, 12].

Serum albumin is a protein synthesized by the liver. Albumin could increase blood volume, maintain plasma colloid osmotic pressure, and combine with many insoluble small molecules in the body to form soluble substances for transport and detoxification. Furthermore, it stabilized globulin and could be used as a nutrient to participate in various reactions. When the human body has severe protein malnutrition or malabsorption, the liver cannot obtain raw materials to synthesize albumin, and its content in the human body decreases. Therefore, albumin could be used as an indicator to reflect low nutritional status. Serum albumin was the strongest clinical predictor of cost [13]. Several studies have jointly demonstrated that low serum albumin level increases mortality and postoperative complications after hip fracture surgery in the elderly [14–16]. Similarly, other studies have shown that serum albumin level was positively correlated with the progression of bony healing, which could be used as an early predictor [17]. Furthermore, studies have shown that serum albumin levels on admission to the emergency department could predict in-hospital complications in elderly trauma patients [18]. Meanwhile, several recent studies have reported the correlation between albumin and DVT after hip fracture. By multivariate analysis, Zuo found that decreased albumin (< 31.7 g/L) was an independent risk factor associated with DVT in bilateral lower extremities after intertrochanteric fracture in the elderly [19]. Zhao also agreed that hypoproteinemia was a risk factor for DVT in geriatric intertrochanteric fractures, although the standard for albumin was less than 35 g/L [20]. Through extensive data analysis, Wang found that patients with albumin < 35 g/ L were associated with an excess risk of DVT after hip fractures [21].

However, the detailed relationship between albumin and DVT after hip fracture was not known, nor was it clear whether there was a nonlinear relationship between them. Therefore, this study aimed to explore the association between albumin and DVT after hip fracture.

## Materials and methods

### Study design

In this retrospective cohort study, we recruited older adults who had a hip fracture from 1 to 2015 to 30 Sep 2019 at the largest trauma center in Northwest China.

This retrospective study was approved by the Ethics Committee of Xi’an Honghui Hospital (No. 202201009). All patients provided informed consent. All human-related procedures followed the 1964 Declaration of Helsinki and its later amendments. The study has been reported according to the STROCSS 2021 guidelines [22].

### Participants

Demographic and clinical data of the patients were obtained from their original medical records. The inclusion criteria were as follows: (1) age ≥ 65 years; (2) X-ray or computed tomography diagnosis of the femoral neck, intertrochanteric, or subtrochanteric fracture; (3) patients who were receiving surgical or conservative treatment in the hospital; (4) availability of clinical data when in the hospital [23]. The exclusion criteria: patients did not receive the anticoagulation treatment.

### Hospital treatment

**Fig. 1:**
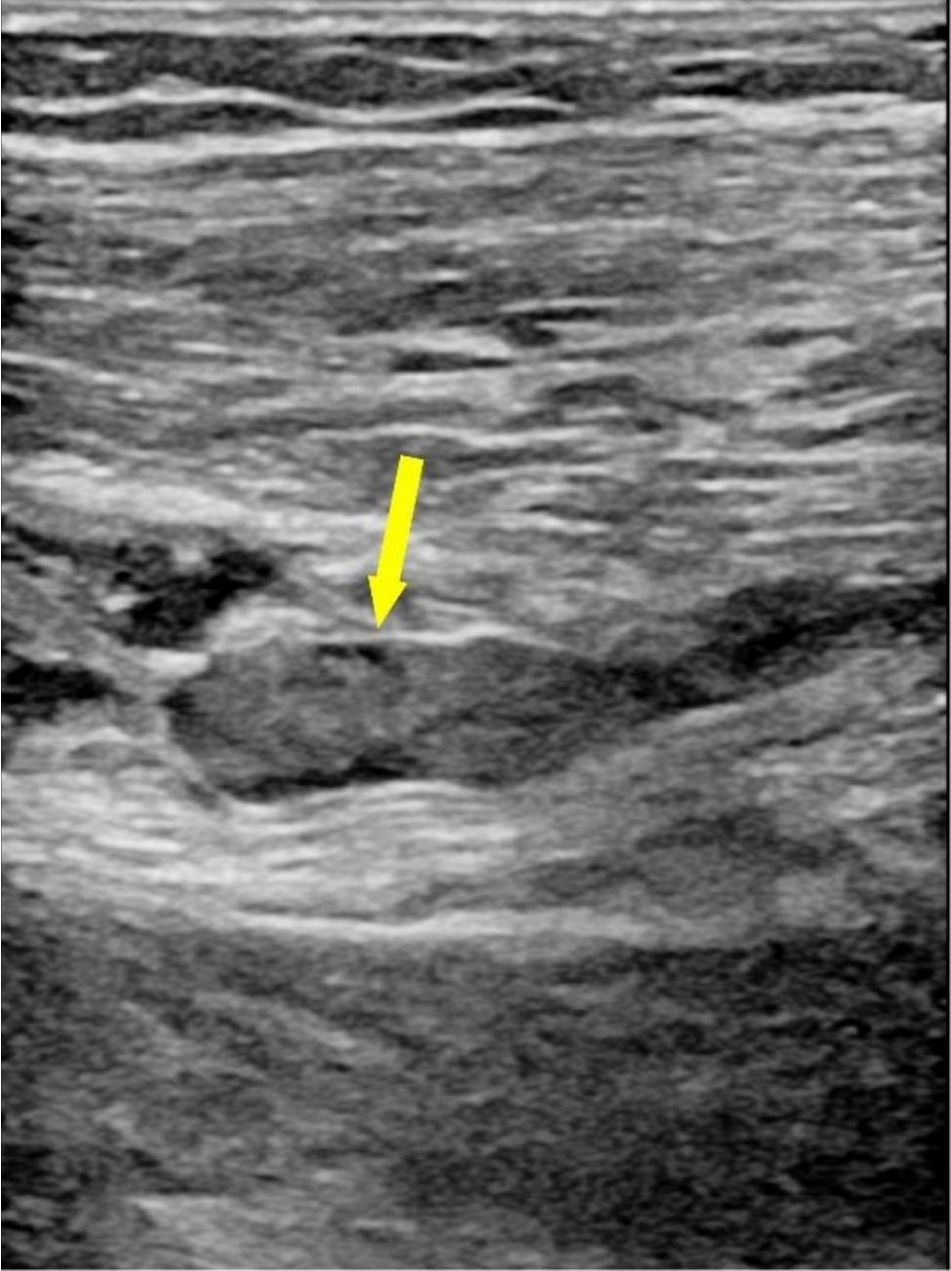
DVT in Doppler ultrasonography (Yellow arrow)

Patients were examined using blood tests to prepare for surgery. Prophylaxis for DVT was initiated at admission. Mechanical thromboprophylaxis (foot intermittent pneumatic compression sleeve, 20 min twice daily) was used to prevent DVT. For patients without contraindications, low molecular weight heparin (Fraxiparine; Glaxo Wellcome Production, GlaxoSmithKline) was injected subcutaneously to prevent DVT [24]. We used Doppler ultrasonography to diagnose the DVT. The diagnostic criteria are the presence of a constant intraluminal filling defect shown in Fig. 1. Patients were examined preoperatively. All patients underwent ultrasonography of bilateral lower extremities on the day before the scheduled surgery [24].

### Endpoint events

The endpoint event in this study was DVT before operation.

### Variables

The dependent variable was preoperative DVT, and the independent variable was the albumin level. Other variables were potentially confounding factors.

### Statistics analysis

Continuous variables are reported as mean ± standard deviation (SD)(Gaussian distribution) or median (min, max)(Skewed distribution), and categorical variables are given as frequencies and percentages. We used χ2 (categorical variables), the One-way ANOVA test (normal distribution), or the Kruskal-Whallis H test (skewed distribution) to test for differences among different albumin (quartile). We divided the patients into four groups to describe demographic and clinical characteristics according to albumin level. We used univariate and multivariate binary logistic regression models to test the connection between albumin and preoperative DVT with three distinct models [24].

All analyses were performed using statistical software packages R (http://www.R-project.org, R Foundation) and EmpowerStats (http://www.empowerstats.com, X&Y Solutions Inc., Boston, MA, USA). Odds ratios (OR) and 95%CI were calculated. A *P*-value < 0.05 (two-sided) was considered to represent statistical significance.

## Results

### Patient characteristics

We enrolled 1819 participants who met the study criteria. The average age was 79.37 ± 6.88 years. There were 550 males and 1269 females. The preoperative albumin was 38.19 ± 4.07 g/L. There were 580 (31.89%) preoperative DVTs, and eight patients were pulmonary embolism. There were two deaths after the operation. There were 12 patients with DVT in proximal veins (femoral and iliac veins), 565 in distal veins (calf muscle, fibular, anterior/posterior tibial, and popliteal veins), and 3 in proximal and distal veins.

Table 1 lists the demographic and clinical characteristics of all 1819 patients and including comorbidities, factors associated with injuries, and treatment strategies.

**Table 1:** The Demographic and clinical characteristics

Albumin quartiles	Q1 albumin level group	Q2 albumin level group	Q3 albumin level group	Q4 albumin level group	*P*-value	*P*-value*
**N**	391	398	461	569		
**Albumin**	32.49 ± 2.42	36.58 ± 0.71	38.92 ± 0.69	42.65 ± 1.96	< 0.001	< 0.001
**Age (year)**	81.62 ± 6.82	80.79 ± 6.56	79.14 ± 6.58	77.02 ± 6.61	< 0.001	< 0.001
**Sex**					0.3	-
Male	124 (31.71%)	130 (32.66%)	140 (30.37%)	156 (27.42%)		
Female	267 (68.29%)	268 (67.34%)	321 (69.63%)	413 (72.58%)		
**Occupation**					0.555	-
Retirement	215 (54.99%)	227 (57.04%)	263 (57.05%)	329 (57.82%)		
Farmer	99 (25.32%)	106 (26.63%)	125 (27.11%)	132 (23.20%)		
Other	77 (19.69%)	65 (16.33%)	73 (15.84%)	108 (18.98%)		
**History of allergy**	18 (4.60%)	17 (4.27%)	16 (3.47%)	24 (4.22%)	0.859	-
**Injury mechanism**					0.471	-
Falling	372 (95.14%)	385 (96.73%)	448 (97.18%)	551 (96.84%)		
Accident	14 (3.58%)	9 (2.26%)	12 (2.60%)	15 (2.64%)		
Other	5 (1.28%)	4 (1.01%)	1 (0.22%)	3 (0.53%)		
**Fracture classification**					< 0.001	-
Intertrochanteric fracture	257 (65.73%)	267 (67.09%)	287 (62.26%)	281 (49.38%)		
Femoral neck fracture	122 (31.20%)	123 (30.90%)	170 (36.88%)	280 (49.21%)		
Subtrochanteric fracture	12 (3.07%)	8 (2.01%)	4 (0.87%)	8 (1.41%)		
**Hypertension**	185 (47.31%)	213 (53.52%)	215 (46.64%)	303 (53.25%)	0.058	-
**Diabetes**	68 (17.39%)	67 (16.83%)	103 (22.34%)	124 (21.79%)	0.075	-
**CHD**	199 (50.90%)	197 (49.50%)	242 (52.49%)	290 (50.97%)	0.856	-
**Arrhythmia**	135 (34.53%)	119 (29.90%)	149 (32.32%)	168 (29.53%)	0.348	-
**Hemorrhagic stroke**	5 (1.28%)	9 (2.26%)	5 (1.08%)	12 (2.11%)	0.428	-
**Ischemic stroke**	134 (34.27%)	113 (28.39%)	155 (33.62%)	167 (29.35%)	0.149	-
**Cancer**	10 (2.56%)	8 (2.01%)	14 (3.04%)	15 (2.64%)	0.825	-
**Multiple injuries**	40 (10.23%)	25 (6.28%)	33 (7.16%)	31 (5.45%)	0.035	-
**Dementia**	29 (7.42%)	11 (2.76%)	20 (4.34%)	13 (2.28%)	< 0.001	-
**COPD**	31 (7.93%)	26 (6.53%)	30 (6.51%)	16 (2.81%)	0.004	-
**Hepatitis**	14 (3.58%)	12 (3.02%)	8 (1.74%)	20 (3.51%)	0.316	-
**Gastritis**	6 (1.53%)	5 (1.26%)	4 (0.87%)	10 (1.76%)	0.658	-
**aCCI**	4.46 ± 1.03	4.27 ± 0.99	4.23 ± 1.13	3.98 ± 1.16	< 0.001	< 0.001
**D-dimer (mg/L)**	5.49 ± 9.54 2.50 (0.16-110.24)	9.03 ± 18.84 4.11 (0.23-194.29)	10.40 ± 22.14 4.90 (0.22-287.42)	12.02 ± 18.75 6.77 (0.16-164.22)	< 0.001	< 0.001
**Time to operation (d)**	4.37 ± 2.72	4.14 ± 2.39	4.15 ± 2.33	4.10 ± 2.35	0.421	0.349
**Time to admission (h)**	168.19 ± 365.19	71.36 ± 162.09	60.38 ± 236.41	50.95 ± 220.73	< 0.001	< 0.001
**Hemoglobin (g/L)**	99.43 ± 19.90	110.15 ± 17.85	115.55 ± 16.18	124.39 ± 15.25	< 0.001	< 0.001
**DVT**	163 (41.69%)	125 (31.41%)	144 (31.24%)	148 (26.01%)	< 0.001	-

Mean + SD/N(%). *P*-value*: For continuous variables, we used the Kruskal Wallis rank-sum test and Fisher’s exact probability test for count variables with a theoretical number of < 10

### Univariate analysis of the association between variates and DVT

We performed univariate analysis to identify potential confounding factors and the relationship between variables and DVT (Table 2). According to the criteria of *P* < 0.1, the following variables were considered in the multivariate binary logistic regression model: sex, fracture classification, multiple injuries, dementia, time to operation, and hemoglobin.

**Table 2:** Effects of factors on DVT measured by univariate analysis

	Statistics	OR (95%CI)	*P*-value
**Age (year)**	79.37 ± 6.88	1.00 (0.99, 1.02)	0.6281
**Sex**			
Male	550 (30.24%)	1	
Female	1269 (69.76%)	1.22 (0.98, 1.52)	0.0731
**Occupation**			
Retirement	1034 (56.84%)	1	
Farmer	462 (25.40%)	1.11 (0.88, 1.40)	0.3963
Other	323 (17.76%)	1.00 (0.76, 1.31)	0.9824
**History of allergy**	75 (4.12%)	1.21 (0.75, 1.96)	0.4355
**Injury mechanism**			
Falling	1756 (96.54%)	1	
Accident	50 (2.75%)	1.01 (0.55, 1.85)	0.9665
Other	13 (0.71%)	2.51 (0.84, 7.51)	0.0993
**Fracture classification**			
Intertrochanteric fracture	1092 (60.03%)	1	
Femoral neck fracture	695 (38.21%)	0.67 (0.55, 0.83)	0.0002
Subtrochanteric fracture	32 (1.76%)	1.87 (0.93, 3.79)	0.0805
**Hypertension**	916 (50.36%)	1.13 (0.93, 1.37)	0.2302
**Diabetes**	362 (19.90%)	0.93 (0.73, 1.19)	0.5771
**CHD**	928 (51.02%)	1.10 (0.90, 1.34)	0.3597
**Arrhythmia**	571 (31.39%)	1.05 (0.85, 1.29)	0.6698
**Hemorrhagic stroke**	31 (1.70%)	1.56 (0.76, 3.20)	0.2295
**Ischemic stroke**	569 (31.28%)	0.88 (0.71, 1.09)	0.2579
**Cancer**	47 (2.58%)	1.22 (0.67, 2.22)	0.5237
**Multiple injuries**	129 (7.09%)	1.49 (1.03, 2.14)	0.0341
**Dementia**	73 (4.01%)	1.61 (1.00, 2.59)	0.0496
**COPD**	103 (5.66%)	0.87 (0.56, 1.35)	0.5364
**Hepatitis**	54 (2.97%)	0.67 (0.36, 1.26)	0.214
**Gastritis**	25 (1.37%)	0.53 (0.20, 1.42)	0.2065
**aCCI**	4.21 ± 1.10	1.00 (0.92, 1.10)	0.9333
**Time to admission (h)**	83.00 ± 256.42	1.00 (1.00, 1.00)	0.2954
**Time to operation (d)**	4.18 ± 2.43	1.06 (1.01, 1.10)	0.0078
**Albumin (g/L)**	38.19 ± 4.07	0.93 (0.91, 0.96)	< 0.0001
**Hemoglobin (g/L)**	113.68 ± 19.41	0.99 (0.99, 1.00)	0.0002
**D-dimer (mg/L)**	9.57 ± 18.36	0.99 (0.98, 1.00)	0.0052

### Multivariate analysis between preoperative albumin and DVT

We used three models (Table 3) to correlate preoperative albumin levels and DVT. When albumin concentration was a continuous variable, linear regression was observed. The fully adjusted model showed a DVT risk decrease of 7% (OR = 0.93, 95% CI: 0.90–0.96), *P* < 0.0001) when albumin concentration increased by one g/L after controlling for confounding factors. When albumin concentration was used as a categorical variable, we found statistically significant differences in the albumin concentration groups of the three models (*P* < 0.0001). In addition, the *P* for trend also showed a linear correlation in the three models (*P* < 0.0001).

**Table 3:** Univariate and multivariate results by linear regression

Exposure	Non-adjusted model	*P*-value	Minimally adjusted model	*P*-value	Fully-adjusted model	*P*-value
**Albumin**	0.93 (0.91, 0.96)	< 0.0001	0.93 (0.91, 0.95)	< 0.0001	0.94 (0.91, 0.97)	0.0002
**Albumin quartiles**						
**Q1**	1		1		1	
**Q2**	0.64 (0.48, 0.86)	0.0028	0.64 (0.48, 0.85)	0.0026	0.71 (0.51, 0.98)	0.0379
**Q3**	0.64 (0.48, 0.84)	0.0016	0.62 (0.47, 0.83)	0.0011	0.69 (0.50, 0.96)	0.0283
**Q4**	0.49 (0.37, 0.65)	< 0.0001	0.47 (0.36, 0.63)	< 0.0001	0.55 (0.39, 0.78)	0.0007
***P *** **for trend**	< 0.0001		< 0.0001		0.0015	

**Data in table**: OR (95% CI) *P*-value

**Outcome variable**: DVT

**Exposed variables**: preoperative albumin

**Minimally adjusted model was adjusted for**: age, sex

**Fully-adjusted model was adjusted for**: age, sex, fracture classification, multiple injuries, dementia, time to operation, hemoglobin, D-dimer

However, we found that the changing interval was slowed down in the Q4 subgroup of albumin concentration (Table 3). This instability indicated the possibility of a nonlinear correlation.

### Curve fitting and analysis of threshold effect

**Fig. 2:**
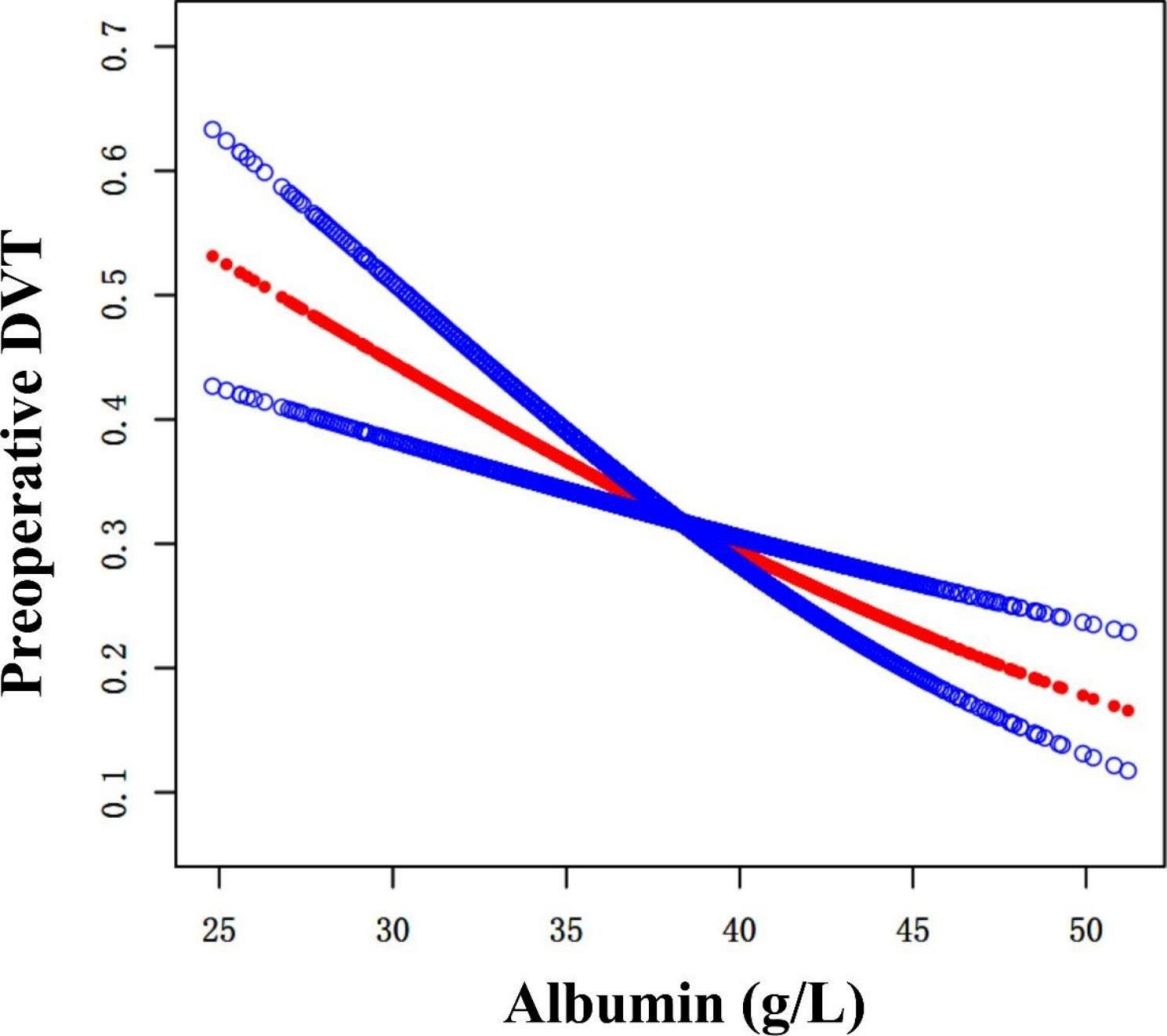
Curve fitting between preoperative albumin and DVT. Adjusted for sex, fracture classification, multiple injuries, dementia, time to operation, hemoglobin, D-dimer

As shown in Fig. 2, there was a linear association between preoperative albumin level and DVT after adjusting for confounding factors. We compared two fitting models to explain this association (Table 4), and we did not observe an inflection point in the model.

**Table 4:** Nonlinearity of preoperative albumin and DVT

Outcome:	OR (95%CI) *P*-value
Fitting model by stand linear regression	0.94 (0.91, 0.97) 0.0002
Fitting model by two-piecewise linear regression	
Inflection point	37.2
< 37.2	0.93 (0.88, 0.98) 0.0116
> 37.2	0.95 (0.90, 1.00) 0.0443
*P* for log-likelihood ratio test	0.631

**Adjusted for** age, sex, fracture classification, multiple injuries, dementia, time to operation, hemoglobin, D-dimer

### Propensity score matching (PSM)

To test the robustness of our results, we performed sensitivity analysis using PSM, as shown in **Figure **S1 and **Table **S1-S3.

There were 1060 patients (58.27%) successfully matched (**Figure **S1; **Table **S1-S3). Some variables did not match the two groups (**Table **S2), including sex, fracture classification, hypertension, ischemic stroke, dementia, gastritis, time to operation, and hemoglobin. We found that the results were stable in the multivariate linear regression results under the PSM and PSM-adjusted models (**Table **S3).

## Discussion

We found a linear association between the serum albumin level and preoperative DVT in geriatric patients with hip fractures. The higher the albumin level, the lower incidence of preoperative DVT. This detailed result showed a DVT risk decrease of 6% (OR = 0.94) when albumin concentration increased by one g/L after controlling for confounding factors. Compared to the Q1 level, Q2, Q3, and Q4 groups could decrease the DVT risk by 29%, 31%, and 45% (OR = 0.71, 0.69, 0.55, respectively). In clinical practice, preoperative albumin concentrations are a predictor of DVT risk.

Several retrospective, prospective studies and systematic reviews have revealed associations between albumin concentration at admission and DVT in geriatric patients with hip fractures in screening the risk factors. A retrospective study by Song et al. reported that a lower albumin level was the independent risk factor for preoperative DVT in 266 patients [25]. Wang et al. reported that reduced albumin was associated with preoperative DVT in a risk prediction model [26]. In a meta-analysis, Wang et al. found that albumin < 35 g/L was a risk factor for preoperative DVT [21]. However, Ding et al. reported that reduced albumin was not associated with preoperative DVT in young and middle-aged patients after hip fracture [27]. In a systematic review and meta-analysis, Kobayashi et al. summarized 3123 Asian patients with hip fractures in 9 studies and found that low albumin was not associated with preoperative DVT [28]. As for this inconsistency results, it might be the age that we consider the most critical factor influencing the outcome because the albumin level varies in different age populations [29]. We have added summary evidence of Table 5 in the association between albumin and DVT. In previous studies [21, 25–28], age is a potential confounding factor and should be divided into different levels. In this study, we only included patients with age > 65 years, and age is not a potential risk factor for preoperative DVT in this study (*P* = 0.6281). Thus, this study included patients with high homogeneity and concluded a clear result for elderly patients ≥ 65 years. In addition, we analyzed the relationship between albumin level and DVT purely and found a stable association. The fully adjusted model showed a DVT risk decrease of 6% when albumin concentration increased by one g/L. However, previous studies only found that albumin was an independent risk factor.

**Table 5:** The summary evidences the association between albumin and DVT.

Year	Author	Study design	Inclusion	Number of subjects	Evaluation primary and secondary outcomes
2022	Wang et al [21]	Meta-analysis	Predictors of preoperative DVT	338 patients	Albumin < 35 g/L was risk factors for preoperative DVT (OR = 1.42)
2022	Zhao et al [20]	Prospective study	Patients aged ≥ 65 years with intertrochanteric fractures	1515 patients	Albumin < 35 g/L was significantly associated with the development of preoperative DVT (OR = 1.516)
2022	Ding et al [27]	Retrospective study	Middle-aged (18–59 years) patients with hip fracture	857 patients	Albumin was **not** an independent risk factor for preoperative DVT
2020	Zue et al [19]	Retrospective study	Elderly patients with intertrochanteric fracture	578 patients	
2022	Xue et al [30]	Retrospective study	Patients aged ≥ 60 years with intertrochanteric fracture	358 patients	Preoperative albumin level was an independent risk factor for preoperative DVT (OR = 2.11)
2022	Zhao et al [31]	Retrospective study	Patients aged 18 years or older; Definite diagnosis of hip fracture and be treated surgically	326 patients	Albumin < 35 g/L was **not** an independent risk factor for preoperative DVT
2022	Song et al [32]	Retrospective study	Closed intertrochanteric fracture patients undergoing surgical interventions	266 patients	Lower level of albumin was the independent risk factors for preoperative DVT (OR = 0.88)
2022	Wang et al [26]	Retrospective study	Patients aged ≥ 60 years with intertrochanteric fracture	855 patients	Albumin < 32.5 g/L was an independent risk factor for preoperative DVT (OR = 2.35)
2023	This study	Retrospective study	Patients aged ≥ 65 years with hip fractures	1819 patients	Albumin level was associated with preoperative DVT (OR = 0.94). The fully adjusted model showed a DVT risk decrease of 6% when albumin concentration increased by 1 g/L.

In addition, the previous studies showed that low serum albumin level was associated with later DVT, and the albumin concentration was divided into a different level, such as < 35 g/L and < 35 g/L [21, 26], < 31.7 g/L and > 31.7 g/L [19]. In Song’s study, they found that the effect size was OR = 0.88 (95%CI: 0.78–0.99; *P* = 0.039) for intertrochanteric fractures. In our study, when we divided the patients into intertrochanteric fracture, femoral neck fracture, and subtrochanteric fracture, we found that the effect size was OR = 0.94 (95%CI: 0.91–0.98; *P* = 0.0065); OR = 0.92 (95%CI: 0.88–0.97; *P* = 0.0017) and OR = 0.93 (95%CI: 0.90–0.96; *P* = 0.0001), respectively. The mean age is 79.37 years in our study, close to 74.5 years in Song’s study [25]. Thus, the effect size in our study is near to Song’s study [25].

Although previous studies have reported a progressive reduction in serum albumin concentration associated with aging [33–35], a meta-analysis showed that the average level of albumin in the community (41.13 g/L) was assessed in older people [36]. However, serum albumin concentration rapidly declined when a hip fracture occurred. In our study, the albumin concentration at admission was 38.19 ± 4.07 g/L. The reason for the acute loss of albumin remains unclear. It is possible that hidden blood loss was the main reason, as described by Liu et al., who reported that an albumin level < 30 g/L at admission was associated with a greater likelihood of more hidden blood loss [37]. According to our results, low albumin concentration was associated with increasing preoperative DVT. In the field of hip fracture, future studies were needed to conduct the randomized controlled trial, showing the effect of albumin supplementation on preoperative DVT.

During the analysis, to explore the actual relationship between albumin and DVT, we not only carried out a linear regression using different adjusted models but also changed the continuous variable of albumin to a categorical variable or performed a trend test for the result. The three models showed a linear association between albumin and preoperative DVT. Furthermore, we also used the PSM method to verify our result in the sensitivity analysis. It matched 1060 patients in PSM analysis, accounting for 58.27% of all patients in this study. Eight variables did not match between the two groups. In the binary logistic regression results under the PSM and PSM adjusted models, we found that the results are the same as those from all the patients. Thus, our results are stable. The PSM exhibited more empirical power than logistic regression [38]. Additionally, we considered confounders that were included in earlier studies, including advanced age [8, 21, 25], sex [9], time to operation [8, 21], hemoglobin [21], dementia [21]. We adjusted the factor of *P* < 0.1 in the univariate analysis, such multiple injuries. Therefore, we comprehensively considered the variables that needed to be adjusted. In addition, we explored the curve association and found no threshold or saturation effect, which supplements the stability of the linear association.

Acutely, this study had some limitations. First, this conclusion applies only to patients aged ≥ 65 because we only included this population in this study. The relationship between albumin and preoperative DVT is unclear for young patients with hip fractures. Second, several factors would increase hypercoagulability. As with a fracture and needing help for the loo, most people reduce their oral intake of food and water. This automatically increases hyper coagulability possibly. We could not collect all these variates because of the nature of the retrospective study, especially the factors before the admission. Thirdly, this study could not confirm the causal relationship between albumin and DVT. In addition, another major limitation of logistic regression was that there was an assumption of linearity between the preoperative DVT and the independent variables.

## Conclusion

The serum albumin is associated with preoperative DVT in geriatric patients with hip fractures, and it could be considered a predictor for the risk of DVT.

### Electronic supplementary material

Below is the link to the electronic supplementary material.

**Supplementary Material 1: Figure S1**. The PSM of two groups under propensity score based on linear model. **Table S1**. Propensity score parameter list. **Table S2**. The balance test of PSM. **Table S3**. Multivariate results by linear regression

Supplementary Material 2

## Data Availability

Xi’an Honghui Hospital implemented the data. According to relevant regulations, the data could not be shared but could request from the correspondence author.

## Declarations

### Registered information

This study is registered on the website of the Chinese Clinical Trial Registry (ChiCTR: ChiCTR2200057323).
