# Supplementary material for: The association between admission serum albumin and preoperative deep venous thrombosis in geriatrics hip fracture: a retrospective study of 1819 patients with age ≥ 65 years
Source: BMC Musculoskelet Disord. 2023 Aug 24;24:672. doi: 10.1186/s12891-023-06776-1 (PMC10464240; doi:10.1186/s12891-023-06776-1)
Supplement: Supplementary file 1 — Supplementary Material 1: Figure S1. The PSM of two groups under propensity score based on linear model. Table S1. Propensity score parameter list. Table S2. The balance test of PSM. Table S3. Multivariate results by linear regression [file 12891_2023_6776_MOESM1_ESM.docx]

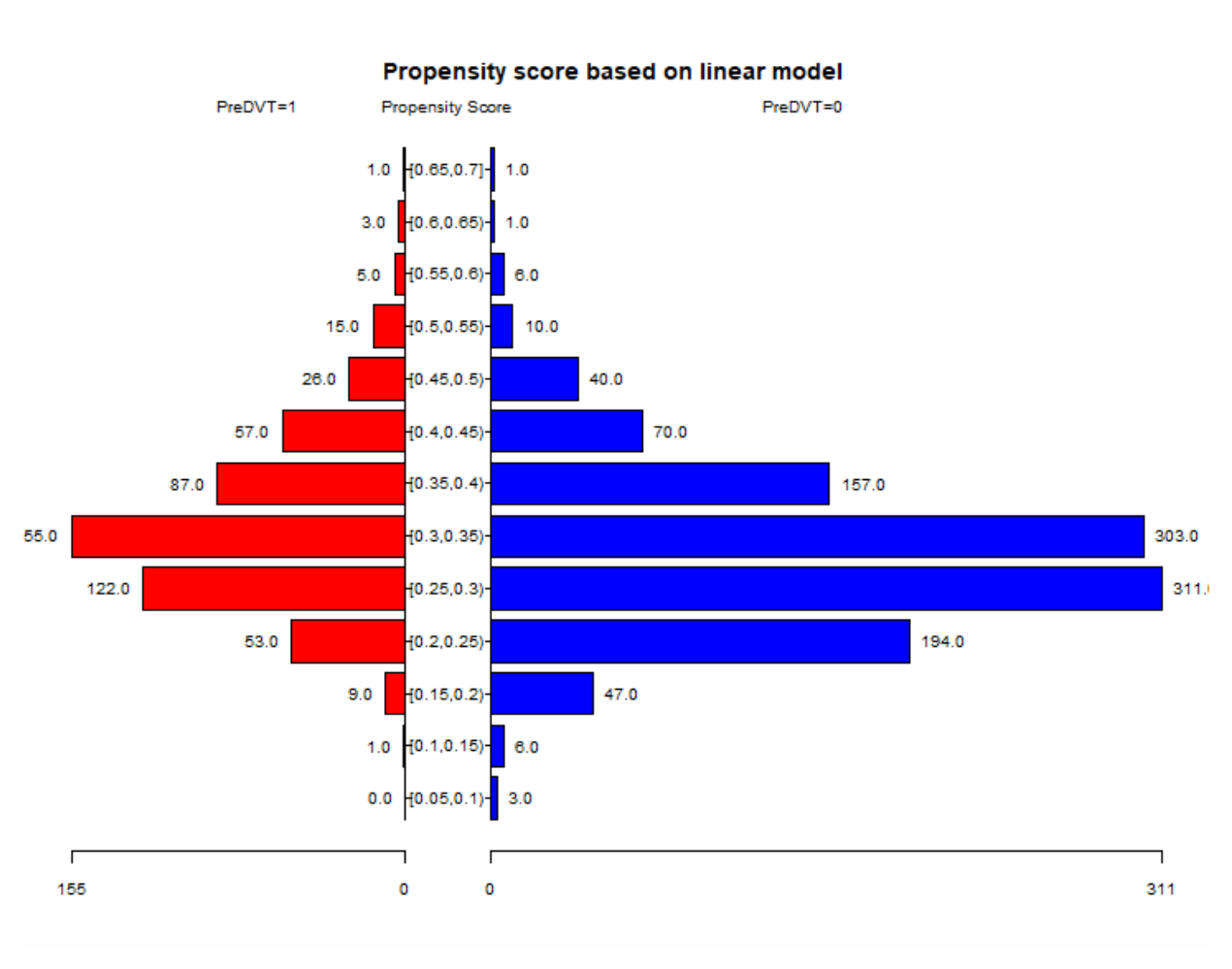


**Figure S1.** The PSM of two groups under propensity score based on linear model.

**Table S1. Propensity score parameter list**

| **The variables used in calculating the propensity score** | **Age, sex, occupation, history of allergy, injury mechanism, fracture classification, hypertension, diabetes, CHD, arrhythmia, hemorrhagic stroke, ischemic stroke, cancer, multiple injuries, dementia, COPD, hepatitis, gastritis, aCCI, time to operation, time to admission, hemoglobin, D-dimer** |
| --- | --- |
| Propensity score algorithm | Multivariate binary logistic regression model |
| C-statistical | 0.6215 |
| Matching method | Greedy matching within specified caliper distances |
| Metric Distances | 0.05 |
| Matching ratio | 1:1 |
| Use of replacement | With replacement |
| Matching sample size | No. of DVT=1: 530 cases  No. of DVT=0: 530 cases  Total 1060 cases |

**Table S2. The balance test of PSM**

| **Variables** | **DVT: No (n=530)** | **DVT: Yes (n=530)** | **Standardized diff.** | ***P* value** |
| --- | --- | --- | --- | --- |
| **Age (year)** | 79.08±7.01 | 79.28±6.50 | 0.0304 | 0.6205 |
| **Sex** |  |  | 0.1843 | 0.0035* |
| Male | 187 (35.3) | 142 (26.8) |  |  |
| Female | 343 (64.7) | 388 (73.2) |  |  |
| **Occupation** |  |  |  | 0.4783 |
| Retirement | 311 (58.7) | 293 (55.3) | 0.0686 |  |
| Farmer | 136 (25.7) | 142 (26.8) | 0.0257 |  |
| Other | 83 (15.7) | 95 (17.9) | 0.0606 |  |
| **History of allergy** |  |  | 0.0090 | 1.0000 |
| No | 505 (95.3) | 506 (95.5) |  |  |
| Yes | 25 (4.7) | 24 (4.5) |  |  |
| **Injury mechanism** |  |  |  | 0.1635 |
| Falling | 514 (97) | 510 (96.2) | 0.0417 |  |
| Accident | 15 (2.8) | 14 (2.6) | 0.0116 |  |
| Other | 1 (0.2) | 6 (1.1) | 0.1167 |  |
| **Fracture classification** |  |  |  | <0.0001* |
| Intertrochanteric fracture | 285 (53.8) | 346 (65.3) | 0.2361 |  |
| Femoral neck fracture | 238 (44.9) | 170 (32.1) | 0.2660 |  |
| Subtrochanteric fracture | 7 (1.3) | 14 (2.6) | 0.0949 |  |
| **Hypertension** |  |  | 0.0302 | 0.6671 |
| No | 262 (49.4) | 254 (47.9) |  |  |
| Yes | 268 (50.6) | 276 (52.1) |  |  |
| **Diabetes** |  |  | 0.0096 | 0.9374 |
| No | 429 (80.9) | 431 (81.3) |  |  |
| Yes | 101 (19.1) | 99 (18.7) |  |  |
| **CHD** |  |  | 0.0377 | 0.5803 |
| No | 265 (50) | 255 (48.1) |  |  |
| Yes | 265 (50) | 275 (51.9) |  |  |
| **Arrhythmia** |  |  | 0.0041 | 1.0000 |
| No | 368 (69.4) | 369 (69.6) |  |  |
| Yes | 162 (30.6) | 161 (30.4) |  |  |
| **Hemorrhagic stroke** |  |  | 0.0584 | 0.4757 |
| No | 523 (98.7) | 519 (97.9) |  |  |
| Yes | 7 (1.3) | 11 (2.1) |  |  |
| **Ischemic stroke** |  |  | 0.1061 | 0.0972 |
| No | 352 (66.4) | 378 (71.3) |  |  |
| Yes | 178 (33.6) | 152 (28.7) |  |  |
| **Cancer** |  |  | 0.0112 | 1.0000 |
| No | 514 (97) | 515 (97.2) |  |  |
| Yes | 16 (3) | 15 (2.8) |  |  |
| **Multiple injuries** |  |  | 0.1485 | 0.0220* |
| No | 503 (94.9) | 483 (91.1) |  |  |
| Yes | 27 (5.1) | 47 (8.9) |  |  |
| **Dementia** |  |  | 0.0843 | 0.2230 |
| No | 512 (96.6) | 503 (94.9) |  |  |
| Yes | 18 (3.4) | 27 (5.1) |  |  |
| **COPD** |  |  | 0.1111 | 0.0937 |
| No | 491 (92.6) | 505 (95.3) |  |  |
| Yes | 39 (7.4) | 25 (4.7) |  |  |
| **Hepatitis** |  |  | 0.0857 | 0.2224 |
| No | 509 (96) | 517 (97.5) |  |  |
| Yes | 21 (4) | 13 (2.5) |  |  |
| **Gastritis** |  |  | 0.1355 | 0.0505* |
| No | 517 (97.5) | 526 (99.2) |  |  |
| Yes | 13 (2.5) | 4 (0.8) |  |  |
| **aCCI** |  |  |  | 0.2181 |
| 2 | 34 (6.4) | 24 (4.5) | 0.0830 |  |
| 3 | 93 (17.5) | 111 (20.9) | 0.0862 |  |
| 4 | 200 (37.7) | 219 (41.3) | 0.0734 |  |
| 5 | 134 (25.3) | 127 (24) | 0.0307 |  |
| 6 | 52 (9.8) | 34 (6.4) | 0.1246 |  |
| 7 | 14 (2.6) | 12 (2.3) | 0.0244 |  |
| 8 | 3 (0.6) | 3 (0.6) | 0.0000 |  |
| **Time to admission (h)** | 73.25±231.75 | 89.57±212.62 | 0.0734 | 0.2325 |
| **Time to operation (d)** | 4.04±2.65 | 4.43±2.60 | 0.1487 | 0.0156* |
| **Hemoglobin (g/L)** | 117.43±19.43 | 111.58±18.72 | 0.3066 | <0.0001* |
| **D-dimer (mg/L)** | 11.45±20.48 | 7.72±11.51 | 0.2245 | 0.0003* |

For continuous variables: (N) Mean±SD, Standardized difference=abs(Mean1-Mean0)/sqrt((S1+S2)/2);

For categorical variables: N (%), Standardized difference = abs(P1-P0)/sqrt((P1*(1-P1)+P0*(1-P0))/2);

* Variables were not successfully matched.

**Table S3. Multivariate results by linear regression**

| **Exposure** | **Fully adjusted model** | **PSM model** | **PSM adjusted model** |
| --- | --- | --- | --- |
| **Albumin** | 0.94 (0.91, 0.97) 0.0002 | 0.92 (0.89, 0.95) <0.0001 | 0.96 (0.92, 0.99) 0.0185 |

**Data in table:** OR (95% CI) *P*-value

**Outcome variable:** DVT

**Exposed variables:** albumin

**Adjust variables in PSM adjusted model:** sex, fracture classification, multiple injuries, gastritis, time to operation, hemoglobin, D-dimer
